# Panner’s disease: literature review and treatment recommendations

**DOI:** 10.1007/s11832-015-0635-2

**Published:** 2015-02-07

**Authors:** Femke M. A. P. Claessen, Jan K. G. Louwerens, Job N. Doornberg, C. Niek van Dijk, Denise Eygendaal, Michel P. J. van den Bekerom

**Affiliations:** 1 Orthopaedic Hand and Upper Extremity Service, Yawkey Center, Massachusetts General Hospital, Harvard Medical School and University of Amsterdam Medical School, 55 Fruit Street, Boston, MA 02114 USA; 2 VU Medical Center Orthopaedic Residency Program (PGY1), Amsterdam, The Netherlands; 3 Orthotrauma Research Center Amsterdam, University of Amsterdam Orthopaedic Residency Program (PGY4), Amsterdam, The Netherlands; 4 Department of Orthopaedic Surgery, Academic Medical Center Amsterdam, Amsterdam, The Netherlands; 5 Shoulder and Elbow Unit Orthopaedic Surgery, Amphia Hospital, Breda, The Netherlands; 6 Shoulder and Elbow Unit Orthopaedic Surgery, Onze Lieve Vrouwe Gasthuis, Amsterdam, The Netherlands

**Keywords:** Osteochondrosis, Panner’s disease, Elbow pain

## Abstract

**Purpose:**

To determine the most up-to-date theory on the aetiology of Panner’s disease, to form a consensus on the assessment of radiographs and to evaluate clinical outcome in order to summarise the best available evidence for diagnosis and treatment.

**Methods:**

A review of studies to date on Panner’s disease. Studies were eligible if: (1) the study provided criteria for defining Panner’s disease in order to eliminate confounding data on other radiographic entities that were mistakenly grouped and presented as Panner’s disease; (2) original data of at least one patient was presented; (3) manuscripts were written in English, German or Dutch; and (4) a full-text article was available. Animal studies, reviews and expert opinions were not included. Because the majority of the studies were case reports, we did not use an overall scoring system to evaluate methodological quality.

**Results:**

Twenty-three articles reporting on Panner’s disease were included. Most cases of Panner’s disease were unilateral in distribution and occurred in boys during the first decade of life. In general, conservative treatment is advised for Panner’s disease. Panner’s disease is a self-limiting disease and the majority of patients heal without clinical impairment.

**Conclusions:**

Based on the results of this review, Panner’s disease should be treated conservatively. Uniform names and descriptions of signs on radiographs would help to make the correct diagnosis. Since Panner’s disease is very rare, higher quality studies are not likely to be performed and, thus, this review provides the best level of evidence on the current knowledge about Panner’s disease.

## Introduction

Osteochondrosis is a term used to describe more than 50 different conditions affecting the immature skeleton. The most frequent site of osteochondrosis in the elbow is the humeral capitellum [1]. In 1927, a Danish orthopaedic surgeon, Dr. Dane Panner, first described radiographic changes of the capitellum in the young adult, subsequently known as Panner’s disease [2–4]. He considered the aetiology of these radiographic changes in the elbow capitellum to be similar to osteochondrosis of the hip epiphysis (Legg–Calvé–Perthes) described 17 years earlier by three orthopaedic surgeons: Arthur Legg, Jacques Calvé and Georg Clemens Perthes [5, 6].

Osteochondrosis and osteochondritis dissecans (OCD) are considered different pathologic entities. Osteochondrosis, defined by irregularity of the humeral capitellum on plain radiographs, occurs shortly after the appearance of the ossific nucleus under 11 years of age, when the cells are considered vulnerable for ischaemia. OCD is described in adolescents and is associated with loose body formation. Panner’s disease is often mistaken for the latter [1]. However, osteochondrosis and OCD have significant differences in aetiology, treatment and outcome [1].

Aetiology, as well as the optimal treatment for Panner’s disease, are subjects of ongoing debate. Therefore, we conducted a systematic review on clinical studies of Panner’s disease with the aims to: (1) determine the most up-to-date theory on aetiology in order to better define these eponyms; (2) to form a consensus on the assessment of radiographs, computed tomography (CT) and magnetic resonance imaging (MRI); and (3) to evaluate patient- and physician-based clinical outcome in order to formulate the best available evidence base for diagnosis and treatment.

## Materials and methods

### Search strategy

To identify studies on Panner’s disease, the following databases (up to June 3, 2014) were searched: EMBASE, MEDLINE vis OvidSP, Web of Science, Cochrane Central, PubMed Publisher, Scopus and Google Scholar (Table 1). The EMBASE search strategy was transferred into similar search strategies for the other databases. References of the included articles were also searched to identify further potentially relevant literature.

**Table 1:**
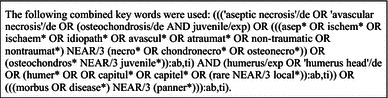
Search strategies

### Study selection

Study selection was assessed by two independent reviewers (FC and JL). Disagreements were solved by an attempt to reach consensus. If no consensus was made, a third reviewer (MB) solved the disagreement. Studies were eligible if: (1) the study provided criteria for defining Panner’s disease in order to eliminate confounding data on other radiographic entities that were mistakenly grouped and presented as Panner’s disease; (2) original data of at least one patient were presented; (3) manuscripts were written in English, German or Dutch; and (4) a full-text article was available. Animal studies, reviews and expert opinions were not included.

### Methodological quality assessment

Two reviewers (FC and JL) independently assessed the methodological quality of all the included studies. Important aspects of methodology were noted: study design, follow-up time and outcomes. Because the majority of the studies were case reports, no pre-printed selection forms or an overall scoring system to evaluate methodological quality was used [7].

### Data extraction

Data extraction was performed by the first independent reviewer (FC) and checked and corrected by the second reviewer (JL). The following data were extracted: study population, patient characteristics, design of study, aetiology, clinical presentation and physical examination, radiological evaluation, follow-up, treatment and outcome measures.

## Results

### Literature search

**Fig. 1:**
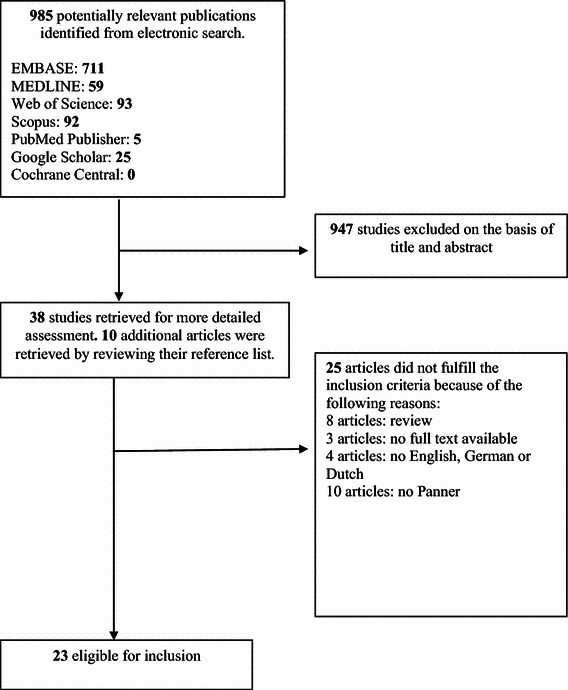
Flow diagram of the study selection and exclusion stages

A total of 23 studies regarding Panner’s disease including 30 patients were included in this review [3–5, 8–27] (Fig. 1). The study and patient characteristics are shown in Table 2. All 23 studies were case reports.

**Table 2:** Study and patient characteristics for Panner’s disease

First author Year Country [Reference]	Study type	No. (% males)	Age, mean (years)	Follow-up (months)	Sports	Aetiology	Symptoms	Imaging	Treatment	Outcome parameters
Krebs 1927 Denmark [4]	Case report	1 (100 %)	8	–	–	Atraumatic	Pain, limitation ROM	X-ray: irregularity capitellum	–	–
Panner 1929 Denmark [3]	Case reports	2 (100 %)	10	10	–	Elbow contusion	Swollen elbow, limitation ROM	X-ray: irregularity capitellum	Conservative	X-ray
March 1944 United States of America [8]	Case report	1 (100 %)	8	<1	Darts	Atraumatic	Swollen elbow, limitation ROM	X-ray: irregularity capitellum	Conservative, rest	Clinical symptoms
Hegemann 1951 Germany [9]	Case report	1 (100 %)	8	5	–	Atraumatic	Pain, limitation ROM	X-ray: irregularity capitellum	Conservative, rest	X-ray
Semmelroch 1952 Germany [10]	Case report	1 (100 %)	9	18	Gymnastics	Elbow contusion	Pain, limitation ROM	X-ray: destruction epiphysis capitellum	Conservative	Clinical symptoms, X-ray
Lange 1954 United States of America [11]	Case report	1 (100 %)	10	24	–	Atraumatic	Pain, stiffness	X-ray: irregularity capitellum, fragmentation capitellum	Conservative, immobilisation	Clinical symptoms, X-ray
Laurent 1956 Finland [12]	Case reports	2 (100 %)	9	59	–	Atraumatic	Pain, swollen elbow, limitation ROM	X-ray: radiotranslucency capitellum, increased density, irregular contour capitellum	Conservative, no strain to elbow + immobilisation	Clinical symptoms, X-ray
Omer 1959 United States of America [13]	Case report	1 (100 %)	8	24	–	Elbow contusion	Pain, swollen elbow, limitation ROM	X-ray: roughening, sclerosis and fragmentation capitellum	Conservative: no strain to arm + immobilisation	X-ray
Heller 1960 United States of America [14]	Case report	1 (100 %)	8	36	–	Elbow contusion	Pain, stiffness, swollen elbow, limitation ROM	X-ray: beginning fragmentation capitellum	Conservative, immobilisation	Clinical symptoms, elbow function, X-ray
Köhne 1961 Germany [15]	Case reports	2 (100 %)	11	14	Sports	Elbow contusion	Pain, limitation ROM	X-ray: irregularity and flattening capitellum, necrosis ossification centre	Conservative, rest, immobilisation	X-ray: regeneration, full recovery
Klein 1962 United States of America [16]	Case report	1 (0 %)	–	28	–	Atraumatic	Pain, swollen elbow, limitation ROM, temperature increase	X-ray: irregularity capitellum, deossification, fragmentation capitellum	Conservative, immobilisation	Clinical symptoms, X-ray
Davidsson 1964 Finland [17]	Case report	1 (100 %)	8	12	–	Atraumatic	Pain, swollen elbow, limitation ROM	X-ray: irregularity, flattening capitellum	Conservative, immobilisation	Clinical symptoms, X-ray
Smith 1964 Scotland [18]	Case reports	2 (100 %)	7	16	–	Atraumatic	Swollen elbow, limitation ROM	X-ray: irregularity capitellum, radiotranslucency capitellum	Conservative, rest, less strain to the elbow	Clinical symptoms, elbow function, X-ray
Bauer 1968 Germany [19]	Case report	1 (100 %)	13	6	–	Elbow contusion	Pain, limitation ROM	X-ray: irregular contour of the humeral capitellum	Conservative, immobilisation	Elbow function, X-ray
Breitkreuz 1968 Germany [20]	Case reports	2 (100 %)	11	6	–	Case 1: elbow contusion Case 2: atraumatic	Limitation ROM	X-ray: irregularity capitellum	Case 1: conservative Case 2: operative, arthroscopic debridement and post-operative immobilisation	X-ray full recovery
Elzenga 1969 The Netherlands [21]	Case reports	3 (67 %)	11	156	Case 2: handball	Case 1: elbow contusion Case 2: sports Case 3: atraumatic	Pain, swollen elbow, limitation ROM	X-ray: irregular contours, fragmentation capitellum, increased density, sclerotic zone	Conservative, no strain to the elbow, immobilisation	Elbow function, X-ray
Maisog 1970 United States of America [22]	Case report	1 (100 %)	15	144	Baseball	Sports	Pain	X-ray: sclerotic, translucent areas, destruction capitellum	–	X-ray
Bouckaert 1973 The Netherlands [23]	Case report	1 (100 %)	6	3	–	Atraumatic	Pain, swollen elbow, limitation ROM	X-ray: sclerotic capitellum	Conservative, immobilisation	Clinical symptoms, X-ray
Mueller 1976 Germany [24]	Case report	1 (0 %)	11	–	Gymnastics	Sports	–	X-ray	–	–
Sty 1978 United States of America [5]	Case report	1 (100 %)	9	–	–	–	Limitation ROM, temperature increase	X-ray: lytic defect in capitellum Bone scan	–	–
Schumacher 1981 Germany [25]	Case report	1 (100 %)	8	12	–	Elbow contusion	Limitation ROM	X-ray: irregularity capitellum, osteolyse	Conservative, immobilisation	Clinical symptoms, elbow function, X-ray
Suman 1982 Scotland [26]	Case report	1 (100 %)	8	26	–	–	Limitation ROM	X-ray: irregularity capitellum	–	Elbow function, X-ray
Stoane 1995 United States of America [27]	Case report	1 (100 %)	9	–	Baseball	Sports	Pain, swollen elbow, limitation ROM	X-ray: sclerosis, irregularity capitellum and fragmentation of the capitellum MRI: decreased signal intensity capitellum, decreased signal and irregularity capitellum	–	–

*ROM* range of motion

### Patient characteristics

All 23 studies described patient characteristics. Of the 30 included patients, 27 were male (90 %). The average age of all the included patients was 9 years (range 6–15 years). There was only study that described Panner’s disease in relation to the dominant arm of the patient [27].

### Aetiology

Twenty-three case reports hypothesised on the aetiology of Panner’s disease [3–5, 8–27]. An elbow contusion in the medical history was mentioned in 13 patients (43 %) [3, 10, 13–15, 19–21, 25], of which four occurred in association with sports activities [10, 15, 19]. Baseball [22, 27], gymnastics [10, 24] and handball [21] are considered to trigger Panner’s disease due to repetitive microtrauma.

### Clinical presentation and physical examination

In 29 patients, the symptoms of Panner’s disease were described [3–5, 8–23, 25–27]. The following symptoms are presented: pain in 19 patients (66 %) [4, 10–17, 19, 22, 23, 27], stiffness in two patients (7 %) [11, 14] and a swollen elbow in 16 patients (55 %) [3, 8, 12–14, 16–18, 21, 23, 25–27]. Twenty-five patients presented limited range of motion [3–5, 8–10, 12–21, 23, 25–27], 18 patients had a limitation of the elbow extension (average 21°; range 10–30) (62 %) [3, 4, 8, 9, 12, 13, 16–18, 20, 21, 26] and seven patients had a flexion deficit (average 23°, range 15–30) (17 %) [4, 8, 17, 18, 20, 21]. In two patients, a warm elbow was described (7 %) [5, 16].

### Radiological evaluation

In all studies, plain radiographs were used for diagnosing Panner’s disease. Irregularity of the humeral capitellum, defined as an irregularity of texture in the epiphysis of the capitellum, was seen in 13 patients (43 %) on a conventional radiograph [3, 4, 8, 9, 11, 15–18, 20, 26, 27]. Irregularity of the texture of the humeral capitellum contour was presented in seven patients (23 %) [12, 13, 19, 21]. Destruction of the epiphysis was reported in one patient (3 %) [10]. An ‘increased density’ of the capitellum was described in five patients (17 %) [12, 21]. Flattening of the humeral capitellum was reported in three patients (10 %) [15, 17]. Klein [16] reported one case of deossification of the capitellum, and Sty and Boedecker [5] and Schumacher et al. [25] described two patients with a lytic defect in the capitellum (10 %). In three case reports, a radiotranslucency of the capitellum was shown (17 %) [12, 18, 22] and fragmentation of the capitellum was seen in six case reports (27 %) [11, 13, 14, 16, 21, 27]. Sclerosis of the humeral capitellum was presented in seven patients (23 %).

The bone scan used in one case report noted increased activity in the humeral capitellum [5]. MRI presented in the case report of Stoane et al. showed a decreased signal intensity of the capitellum on T1 series. Decreased signal and cortical irregularity, as well as high signal in the joint space consistent with a joint effusion, were also seen on T1 series. Joint effusion shows high signal on T2 [27].

### Treatment

Seventeen case reports described the treatment for Panner’s disease [3, 8–21, 23, 25]. In six patients, rest was advised (26 %) [8, 9, 15, 18]. No case reports described the recommended duration of rest. Refrain from strenuous arm activities, such as pitching, baseball and carrying heavy items, was advised in five case reports (30 %) [12, 13, 18, 21].

Immobilisation was preferred in 16 patients (53 %) [11–17, 21, 23, 25]. In nine patients, a cast was recommended (30 %) [11, 15, 17, 19, 21, 23]. A cast was recommended for an inconsistent period of time ranging from 4 weeks to 11 months.

Omer and Conger [13] used a sling for 5 days, and Laurent and Lindstrom [12] used a bandage for 1 month. In five patients, the use of a splint for the treatment of Panner’s disease was recommended [14, 21, 25]. Heller and Wiltse described the use of a splint in 120° of elbow flexion for 3 weeks full time and then 6 months during the day [14], Elzenga [21] advised to use the splint for 4 weeks and Schumacher et al. [25] for 1 year.

Breitkreuz [20] reported arthroscopic debridement and a post-operative cast for 4 months as treatment for Panner’s disease. Smith [18] mentioned the use of non-steroidal anti-inflammatory drugs for pain relief.

### Functional and radiographic outcome

Nineteen case reports described standardised outcome measurements [3, 8–23, 25, 26]. Radiographic reports were used as an outcome measure in 17 case reports [3, 9–23, 25, 26]. Full recovery and complete healing of the capitellum was seen in ten case reports (37 %) [8–10, 12, 15, 16, 20, 22, 23, 26]. Almost complete recovery was seen in 14 patients (56 %) [3, 11, 13, 14, 17–19, 21, 25]. Some irregularity and flattening of the capitellum, or sclerosis in the capitellum, was still visible on some radiographs [14, 17, 18, 21, 25].

Subjective clinical symptoms were used as post-treatment outcome measurement in 11 patients [10–12, 14, 16–18, 23, 25]. Nineteen patients described pain at the end of treatment [4, 10–17, 19, 22, 23, 27]. Objective elbow function was used as an outcome measurement in nine patients [11, 14, 18, 19, 21, 25, 26]. A full range of motion was described in seven patients (78 %) [12, 19, 22, 26], 20° of flexion contracture in one patient [19] and a loss of the terminal 5° of both flexion and extension in one patient [14].

## Discussion

Panner’s disease is defined as an osteochondrosis of the humeral capitellum [25]. Some experts suggest that Panner’s disease and OCD of the humeral capitellum might be a continuum of disordered endochondral ossification, depending on the age and severity of the lesion [28, 29]. However, evidence is seen for two separate diseases, because of the difference in age of presentation, radiographic findings and prognosis [1]. In general, patients aged 10 years and younger have lesions similar to those described by Dane Panner, without intra-articular loose bodies [28, 29].

In our review, most patients with Panner’s disease were boys (90 %). It is believed that Panner’s disease predominantly occurs in boys because of the delayed appearance and maturation of the secondary growth centres [30]. The higher risk for traumatic injuries in boys could be another explanation for the increased prevalence of Panner’s disease in boys, as half of the cases in our review reported a precedent trauma.

Valgus stress in throwing athletes and increased axial load to the radiocapitellar joint in gymnasts can typically result in lateral compression injuries of the elbow. Lateral compression injuries can lead to several lesions, including Panner’s disease and OCD of the humeral capitellum.

Several experts believe that abnormal valgus stress after the age of 5 years is the most important factor in the development of Panner’s disease [28–31]. The capitellum has a rich vascular supply prior to the age of 5 years. Afterwards, the nucleus of the capitellum is mainly supplied by posterior vessels functioning as end arteries [32]. If those vessels are disrupted by repetitive stress (i.e. throwing), ischaemia can develop [30, 31]. This may result in the disordered endochondral ossification [29, 32] called Panner’s disease.

Most patients with Panner’s disease presented with a history of several weeks of pain and stiffness in the elbow, often with a history of valgus stress. Symptoms were increased by activity and relieved by rest by most patients [1]. A small effusion and swelling may be noted. Limited range of motion is typically observed with approximately 20° of extension loss and, less commonly, loss of flexion [2]. The duration of symptoms varied from a few months to 2 years.

No evidence for a correlation between radiographic parameters and symptoms was found. Epiphyseal and contour irregularity of the humeral capitellum are often observed. Fragmentation of the capitellum, radiotranslucent areas and sclerosis were also often documented. Fusion between the centre of ossification of the capitellum and the adjacent centres occurs roughly at the age of 10 years in girls and at the age of 12 years in boys. Panner’s disease can develop during this period  [11, 32]. The radiological improvement occurs over 1–3 years [21, 33]. Studies to date agree that osteochondrosis passes through stages, similar to Perthes’ disease [17]. With a bone scintigraphy, changes in vascularity and osteogenesis can be measured, but it cannot distinguish between Panner’s disease and other diseases that change the vascularity and osteogenesis (i.e. rheumatoid arthritis) [5]. In lumbar osteochondrosis, the degree of disc prolapse shown on CT is correlated to the intensity of neurological symptoms [34]. However, there is no evidence for a role of CT in diagnosing Panner’s disease. MRI has been effectively used for diagnosing Perthes’ disease and avascular necrosis. Even though MRI is more costly, it could also be useful in diagnosing Panner’s disease [27]. A decreased signal intensity of the capitellum is seen on a T1 series [27] and an increased signal intensity is shown on a T2 series [35].

Panner’s disease is frequently used as a term to describe osteochondrosis, OCD and osteonecrosis of the elbow. We recommend the use of one name for Panner’s disease instead of several different terms to make the diagnosis more uniform and to reduce misdiagnoses. Preferably this name is not an eponym, but a description of the most frequently encountered finding on MRI or radiographs, for example osteochondrosis of the humeral capitellum. Also, terms used to describe radiographs should be the same. In the studies discussed in our article, a lytic defect in the capitellum, radiotranslucency of the capitellum and deossification of the capitellum could be similar signs on radiographs, but due to the different descriptions, it is not clear.

Panner’s disease is probably underdiagnosed, because the symptoms and findings on radiographs can be subtle [13, 27]. In some cases in this review, OCD could be misnamed Panner’s disease.

In general, conservative treatment is advised for Panner’s disease. Reduction of elbow activities that increase valgus stress may relieve pain and allow a return to normal elbow motion and function. Immobilisation and anti-inflammatory medications, such as non-steroidal anti-inflammatory drugs, provide marked relief in most cases [18].

Panner’s disease is a self-limiting disease and the majority of patients heal without any morbidity.

There were several limitations to the included studies of this systematic review. This review is mainly based on case series and, therefore, the strength of evidence is limited by the quality of the available studies. Secondly, several quality criteria are not clearly described; specifically, information on potential bias (e.g. inclusion bias), handling of missing data and reasons for dropout were lacking in most studies. Several different names and descriptions are used for Panner’s disease. Therefore, some patients could be diagnosed as Panner’s disease, while they have OCD of the humeral capitellum or traumatic epiphyseal damage instead.

Based on this review, we recommend that Panner’s disease should be treated conservatively.

Uniform names and description of radiographic signs for Panner’s disease would help to reduce misdiagnoses. Future studies on Panner’s disease should investigate the possible correlation between radiographic appearance and symptoms. Furthermore, the duration of conservative treatment options should be compared.

However, since this disease is very rare, higher quality studies are not likely to be performed and, thus, this review, although limited by the quality of included studies, provides the best level of evidence on what is known about Panner’s disease.
